# Copper Redox Cycling Inhibits Aβ Fibre Formation and Promotes Fibre Fragmentation, while Generating a Dityrosine Aβ Dimer

**DOI:** 10.1038/s41598-018-33935-5

**Published:** 2018-11-01

**Authors:** Miao Gu, David C. Bode, John H. Viles

**Affiliations:** School of Biological and Chemical Sciences, Queen Mary, University of London, Mile End Road, London, E1 4NS UK

## Abstract

Oxidative stress and the formation of plaques which contain amyloid-β (Aβ) peptides are two key hallmarks of Alzheimer’s disease (AD). Dityrosine is found in the plaques of AD patients and Aβ dimers have been linked to neurotoxicity. Here we investigate the formation of Aβ dityrosine dimers promoted by Cu^2+/+^ Fenton reactions. Using fluorescence measurements and UV absorbance, we show that dityrosine can be formed aerobically when Aβ is incubated with Cu^2+^ and hydrogen-peroxide_,_ or in a Cu^2+^ and ascorbate redox mixture. The dityrosine cross-linking can occur for both monomeric and fibrillar forms of Aβ. We show that oxidative modification of Aβ impedes the ability for Aβ monomer to form fibres, as indicated by the amyloid specific dye Thioflavin T (ThT). Transmission electron microscopy (TEM) indicates the limited amyloid assemblies that form have a marked reduction in fibre length for Aβ(1–40). Importantly, the addition of Cu^2+^ and a reductant to preformed Aβ(1–40) fibers causes their widespread fragmentation, reducing median fibre lengths from 800 nm to 150 nm upon oxidation. The processes of covalent cross-linking of Aβ fibres, dimer formation, and fibre fragmentation within plaques are likely to have a significant impact on Aβ clearance and neurotoxicity.

## Introduction

Alzheimer’s disease (AD) is the most common form of dementia which directly affects more than 30 million people worldwide^1^. The amyloid cascade hypothesis has been widely accepted as an explanation for AD pathology^2^. Central to the hypothesis is the accumulation of amyloid-β peptide (Aβ) into fibrillar plaques within the brain interstitium^3^. However, it is the self-assembly of Aβ into dimers and small oligomers that confers toxicity^4–10^. In addition to the formation of Aβ assemblies, AD is characterised by the presence of numerous markers of oxidative stress^11–13^. Patients with AD are reported to have increased levels of oxidative damage in the brain tissue, as well as in the cerebrospinal fluid (CSF) and plasma. Observed oxidative damage includes the oxidative modifications of proteins^14^ which include Aβ^15–17^, DNA and RNA oxidation^18^, and also lipid peroxidation^19^. This is particularly apparent in sporadic AD where aging leads to a reduction in antioxidant defences in the brain^20^. Interestingly, these oxidative markers can be observed before plaque formation^21,22^. Another feature of AD pathology is the presence of concentrated metal ions in plaques, and disrupted metal ion homeostasis^15,23–25^. In particular, redox active copper ions are linked to AD phenotypes in *drosophila*^26,27^ and rabbit models^28^.

Several residues within Aβ, in particular Methionine, Tyrosine, and Histidine have been shown to be oxidized both *in vitro* and within plaques of patients with AD^15–17,29–32^. A proportion of Aβ has its single methionine (Met35) oxidized to a sulphoxide within Aβ plaques^15,33^, and the Met oxidation can impact Aβ fibre formation^34,35^. In addition to methionine oxidation, tyrosine can also be oxidised to form a dityrosine covalent dimer. Immuno-gold-labelling provides good evidence for co-localisation of dityrosine and Aβ within AD plaques^17^, while mass-spectrometry has revealed the presence of dityrosine cross-linked Aβ in the brains of AD patients^32^. Furthermore, dityrosine concentrations are five- to eight-fold higher in the hippocampus and neocortical regions of the AD brain^36^. Taken together these observations constitute compelling evidence of Aβ dityrosine cross-linking within the AD brain^17,32,36^.

**Figure 1:**
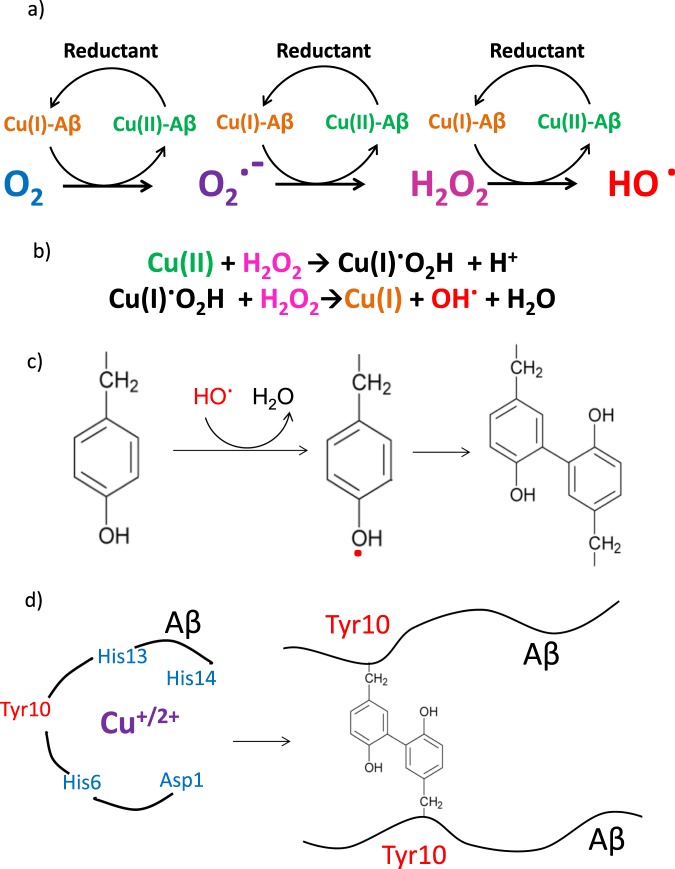
(**a**) Fenton reactions: The redox cycling of Cu(II/I) bound to Aβ in aerobic conditions results in the production of superoxide; hydrogen peroxide; and the hydroxyl radical. The reductant used in this study was ascorbate (**b**) Haber-Weiss cycle: Hydrogen peroxide will also produce Cu(I) and the hydroxyl radical via the Haber-Weiss cycle. (**c**) Dityrosine formation: The hydroxyl radical can generate a tyrosine radical that then combines with tyrosine to form dityrosine, the covalent bond typically forms at the C_ortho_-C_ortho_ position on the ring, although the C_meta_ can also occur. (**d**) Covalent Aβ dimer: Cu(II/I) coordination to Aβ centred at the His imidazole rings produces hydroxyl radicals close to the Tyr10, that will form a covalent dimer.

Oxidation of methionine can be caused by H_2_O_2_ alone, a ubiquitous molecule *in vivo*^37^, without the presence of oxidative free-radicals^30,34,38^. In contrast, dityrosine formation requires the presence of a radical^29,39,40^. The most commonly reported pathway for dityrosine formation occurs when a tyrosine radical, reacts with a second tyrosine to form a covalent cross-link, Fig. 1^40–42^. A dityrosine Aβ(1–16) dimer (but not full-length Aβ) has also be described by mass-spectrometry^43^. The tyrosine radical can readily be generated by highly reactive hydroxyl radicals, produced by Fenton redox cycling of iron as haem^44^ or copper ions^45^, see Fig. 1. Cu^2+^ has a sub-nanomolar affinity for Aβ with a consensus conditional dissociation constant at pH 7.4 reported in the 50–100 pM range for Aβ(1–40) and Aβ(1–42) (for monomer and fibre)^46^ and Aβ(1–16)^47,48^. Cu(II) is also found bound to Aβ in plaques^15,23,24^. Cu^2+^ has an even tighter femto-molar affinity for truncated forms of Aβ(11–40) and Aβ(4–40) which are capable of co-fibrillising with full-length Aβ, and are also found at appreciable levels in plaques^49,50^. The affinity of Cu^+^ for Aβ is less well established, but the most recent affinities reported for Aβ(1–16) are also sub-nanomolar; 40 pM at pH 7.4^47^.

Cu^2+^ and Cu^+^ coordinates to Aβ via histidine residues (His6, His13 and His14) which are close to the Tyr10 side-chain^46,51–55^. Specifically, the pH sensitive tetragonal Cu^2+^ complex involves interchangeable coordination from two of the three histidine imidazole nitrogen’s (His6, His13 and His14) together with coordination at the N-terminal Asp1^46,47,51,55^. Whereas, the Cu^+^ complex comprises interchangeable pairs of imidazole nitrogens, to form a linear complex^47,52–55^. An “in-between” state responsible for ROS production has been proposed which involves Asp1, and a single histidine imidazole nitrogen^54,55^. Importantly, Cu^2+^ is redox active in the Cu(Aβ) complex and readily generates hydroxyl radicals, superoxide and H_2_O_2_ in the presence of a physiological reductant such as ascorbate or in a Cu + H_2_O_2_ Haber-Weiss redox system, highlighted in Fig. 1^30,56–61^.

Small oligomers, and in particular Aβ dimers, have been implicated as the neuro-toxic forms of Aβ^4,10^, rather than larger Aβ fibre assemblies^4–8^. Aβ dimers are shown to be elevated in AD patients’ blood^62^. Insoluble amyloid plaque cores taken from the AD patients’ cerebral cortex do not inhibit long-term potentiation in mice, unless they are solubilised to release Aβ dimers^4^. Furthermore, Aβ toxicity in primary cell-culture has been demonstrated to decrease when Tyr radical production is inhibited by spin trapping^40^. This study went on to show that an Aβ analogue which lacks tyrosine at position 10 did not induce cytotoxicity^40^. Taken together with more recent studies, this suggests that the covalently cross-linked dityrosine Aβ dimer may have an important role in Aβ neurotoxicity^40,63^. Notably, a designed disulphide linked Aβ dimer Aβ(Ser26Cys) has also been shown to be synaptotoxic^4^.

Despite the presence of dityrosine in plaques^17,32^, and the role for Aβ dimers in neurotoxicity^4,10^ there have been relatively few studies describing the effect of dityrosine formation on Aβ fibre assembly. Some studies have suggested that dityrosine formation might accelerate fibre formation, with stable dimer formation being the first step in fibre assembly^42^, as has been reported for other covalent dimers of Aβ^64,65^. However, more recent studies suggest dityrosine formation will inhibit fibre assembly and promote formation of oligomer and protofibrillar assemblies^63,66–68^. In particular, the process of Copper and Hydrogen peroxide Induced Cross-linking of Unmodified Proteins (CHICUP), can stabilize large SDS resistant Aβ oligomers, attenuate fibril formation, and cause prolonged disruption to biomimetic lipid vesicles^66^. In related studies, oxidative stress and dityrosine cross-linking of α-synuclein has also been implicated in Parkinson’s disease^69,70^.

Here we have investigated the effect of dityrosine formation on Aβ self-assembly. We chose to use copper ions to oxidize Aβ and generate dityrosine in these investigations, as it is thought to be a source of dityrosine formation *in vivo*^11,39–42^. Using dityrosine fluorescence and UV absorbance, we have monitored dityrosine production rates for monomeric and fibrillar Aβ. Using two aerobic oxidizing systems, Copper + H_2_O_2_ or Copper + ascorbate, we have investigated the impact that each system has on Aβ fibre assembly. Our *in vitro* studies on Aβ oxidation and dityrosine formation indicate that rather than accelerate amyloid fibre formation, Aβ assemblies are largely protofibrillar, while fibres become fragmented.

## Results

### Dityrosine Formation for Aβ monomers and fibres

We chose a combination of Cu^2+^ with H_2_O_2_ under aerobic conditions to oxidize Aβ, because Cu^2+^ is found concentrated in Aβ plaques^15,23–25^ while H_2_O_2_ is a physiologically common and relatively mild oxidant^37^. To determine the extent by which this redox system is capable of producing dityrosine within Aβ, we used fluorescence spectroscopy. Dityrosine produces a characteristic intrinsic fluorescence signal with an emission at 410 nm when excited at 310 nm^17,29,40,71–73^.

**Figure 2:**
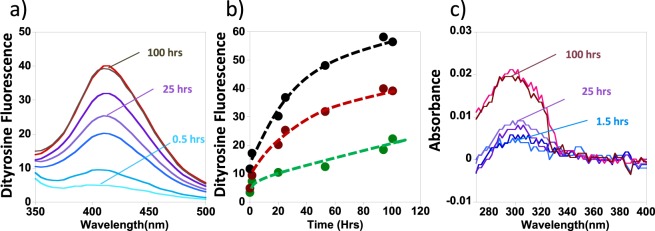
Cu-Aβ(1–40) in the presence of hydrogen peroxide will generate dityrosine, monitored by fluorescence at 410 nm. (**a**) Fluorescence spectra of monomeric Aβ(1–40) with 5 µM Cu^2+^ and 800 µM H_2_O_2_ over 100 hours, fluorescence excitation at 310 nm. (**b**) Increase in dityrosine fluorescence at 410 nm with time, 5 µM Cu^2+^ and 400 µM H_2_O_2_ (green), 5 µM Cu^2+^ and 800 µM H_2_O_2_ (red), and 5 µM Cu^2+^ and 1600 µM H_2_O_2_ (black). (**c**) UV absorbance spectra of Aβ(1–40) over time, difference spectra shown with spectra for un-oxidised Aβ subtracted from each spectra. 5 µM Cu^2+^ and 800 µM H_2_O_2_. 10 µM Aβ(1–40) are incubated at pH 7.4, 100 µM HEPES buffer and 160 mM NaCl.

Figure 2a shows the fluorescence spectrum of Aβ(1–40) monomer evolving with time as it is incubated with a Cu^2+^ + H_2_O_2_ oxidizing system. Aβ(1–40) (10 µM) was incubated with Cu^2+^ (5 µM), and three levels of H_2_O_2_ (0.4, 0.8, 1.6 mM). The fluorescence emission signal at 410 nm was then monitored over time. The data presented in Fig. 2b indicates that the greater concentrations of H_2_O_2_ cause increased amounts of dityrosine to form more rapidly. After 100 hours, the increase in fluorescence signal starts to plateau.

We wanted to quantify the amount of dityrosine that the Cu^2+^  + H_2_O_2_ + O_2_ system can generate. Fluorescence spectra cannot readily be used for direct quantitation, however, dityrosine has a UV absorbance band at 315 nm, with a known extinction coefficient, ε_*315 nm*_ = 5,000 M^−1^cm^−1^ at pH 7.5^71,72^, which was used to quantify the amount of dityrosine formed. Figure 2c shows a UV difference absorption spectrum of oxidized Aβ(1–40), with the absorbance spectrum for unoxidized Aβ subtracted from all spectra. As Aβ(1–40) is oxidized over time, increasing amounts of dityrosine absorbance are observed. After 100 hours incubation with Cu^2+^ (5 µM) and H_2_O_2_ (800 µM), an absorbance at 315 nm of 0.018 was recorded. The weak absorbance signal at 315 nm is a shoulder on the main tyrosine absorbance band at 280 nm, and so this method of dityrosine quantification can only be approximate. An accuracy of 0.005 absorbance units equates to a dityrosine concentration of 3.6 +/− 1.0 µM (Aβ dimers). This absorbance value indicates that between 50–90% of Aβ(1–40) has formed dityrosine.

In order to further investigate the conversion of tyrosine to dityrosine, we used Cu^2+^  + H_2_O_2_ to oxidize Aβ(1–16). The N-terminal fragment still retains the Tyr at position 10, and the Cu^2+^ binding ligands, but is less prone to aggregation and consequently the absorbance spectra are less affected by light scatter. According to the absorbance spectrum, Supplemental Fig S1, incubation with Cu^2+^ and H_2_O_2_ (1 mM) for 120 hours will convert between 50–90% of the tyrosine to dityrosine, producing comparable fluorescence intensity as is observed for monomeric Aβ(1–40), in Fig. 2.

We wanted to use a second method to quantify the amount of dityrosine formed. SDS-PAGE was able to monitor dimer formation over time. In the presence of Cu^2+^  + H_2_O_2_ the intensity of the band for Aβ(1–40) monomer decreased with time as the band for the Aβ dimer appears, Supplemental Fig S2. Quantification of the band intensities indicates the rate of conversion of monomer to dimer, to closely agree with the fluorescence and UV absorbance measurements. In particular, after 100 hours incubation approximately 50% of the Aβ(1–40) monomer has formed a covalent dimer.

**Figure 3:**
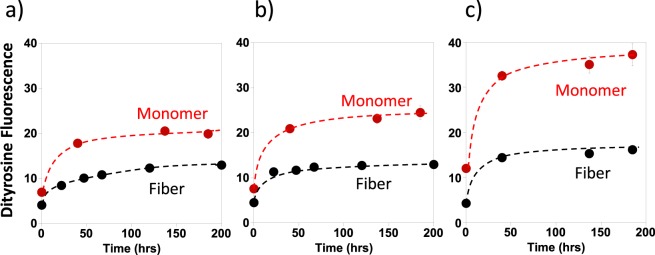
Comparison of dityrosine formation rates for monomeric and fibrillar Aβ(1–40). Both Aβ monomer and fibre can be oxidized to form dityrosine using Cu^2+^ + H_2_O_2_; but Aβ(1–40) monomer forms dityrosine more readily. Dityrosine fluorescence (410 nm) was monitored over 200 hrs for both 10 µM Aβ(1–40) monomer (red) and fibre (black) at a range of Cu + H_2_O_2_ concentrations: (**a**) 5 µM Cu^2+^  + 200 µM H_2_O_2_; (**b**) 10 µM Cu^2+^  + 200 µM H_2_O_2_; (**c**) 5 µM Cu^2+^  + 400 µM H_2_O_2_. All samples contain 100 mM HEPES buffer at pH 7.4 and 160 mM NaCl.

Monomeric Aβ can therefore readily form a dityrosine cross-link, next we investigated whether dityrosine can also be formed when Aβ is in its fibril form. Figure 3 directly compares the rate at which dityrosine forms within Aβ(1–40) monomer compared to that of Aβ(1–40) fibres; at three different levels of H_2_O_2_ + Cu^2+^. The dityrosine formation largely plateaus after 50–100 hours of incubation. The total amount of dityrosine fluorescence is consistently less by a factor of two, for Aβ fibres compared to the same oxidising condition for monomeric Aβ.

We were also interested in how the relative concentration of H_2_O_2_ + Cu^2+^ affects the total amount of dityrosine produced. With a fixed amount of Cu^2+^ present (5 µM), increasing the concentration of H_2_O_2_ from 0.4 mM to 1.6 mM promotes dityrosine formation (Fig. 2b). However, on using the same concentration of H_2_O_2_ (0.4 mM) while doubling the Cu^2+^ concentration from 0.5 molar equivalents to equimolar concentrations, there is a minimal impact on the total amount of dityrosine produced (Fig. 3a,b). The copper does not become depleted in the Fenton and Haber-Weiss reactions, so doubling the levels of Cu^2+^ present does not strongly impact the total amount of dityrosine formed.

To summarize, the Cu^2+^ + H_2_O_2_ redox cycling system is capable of oxidizing most (between 50–90%) of the Aβ monomer within 100 hours to produce the dityrosine dimer. Both Aβ(1–40) monomer and fibre can form considerable amounts of dityrosine, but with the Cu^2+^ + H_2_O_2_ system, dityrosine formation appears more readily achieved for Aβ monomers.

### Dityrosine formation impedes Aβ fibre assembly

As dityrosine is reported to co-localize within Aβ plaques *ex-vivo*^17^, we wanted to probe the possibility of dityrosine formation as a mechanism to promote fibre assembly. In order to study the effect of dityrosine formation on the kinetics of amyloid-β fibre growth, we used a 96-micro-wellplate assay, in which the binding of Thioflavin T (ThT) to Aβ fibres induces ThT fluorescence at 490 nm and so can be used to monitor the kinetics of fibre formation. Different amounts of Cu^2+^ and H_2_O_2_ were added to Aβ(1–40) monomer and the development of a ThT signal was monitored.

**Figure 4:**
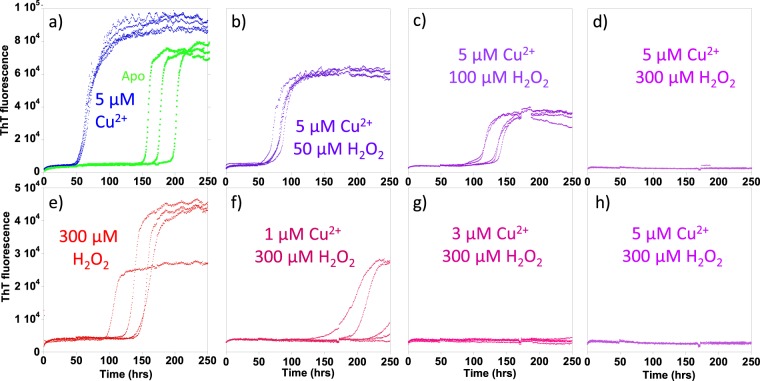
The effect of Aβ(1–40) oxidation on the rates of fibre assembly. Redox cycling of Cu^2+/+^ oxidizes Aβ and inhibits its formation of fibres, causing extended lag-times and reduced total fibre mass. ThT fluorescence was used to monitor Aβ fibre formation in the presence of 5 µM Cu^2+^ and increasing levels of H_2_O_2_ (**a**–**d**). (**a**) 0 µM H_2_O_2_; (**b**) 50 µM H_2_O_2_; (**c**) 100 µM H_2_O_2_ and (**d**) 300 µM H_2_O_2_. While (**e**–**h**) has 300 µM H_2_O_2_ with increasing levels of Cu^2+^. (**e**) 0 µM Cu^2+^; (**f**) 1 µM Cu^2+^; (**g**) 3 µM Cu^2+^ and (**h**) 5 µM Cu^2+^. Fibre formation is also shown for Aβ(1–40) alone (green traces). 10 µM Aβ(1–40) was incubated with 100 mM HEPES, pH 7.4, 20 µM ThT and 160 mM NaCl at 30 °C with intermittent agitation.

Fibre formation is strongly inhibited by conditions that cause dityrosine formation, Fig. 4. Cu^2+^ alone accelerates Aβ(1–40) fibre formation (Fig. 4a and Supplemental Fig S3a) as previously reported^74,75^, but co-incubation with H_2_O_2_ causes significant inhibition of fibre generation. Sub-stoichiometric Cu^2+^ (5 µM) incubated with 100 µM H_2_O_2_ decreases the maximum ThT signal by 50% while the lag-phase of fibre formation increases from 70 to 125 hours, while higher concentrations of H_2_O_2_ with Cu^2+^ completely inhibit fibre formation (Fig. 4a–d).

Similar effects were observed when Aβ was incubated with 300 µM H_2_O_2_, and varying amounts of Cu^2+^ present, Fig. 4e–h. For example, 300 µM H_2_O_2_ reduces the total amount of fibres formed while accelerating their formation a little (Fig. 4e and Supplemental Fig S3b), however upon the addition of even small amounts of Cu^2+^ (1 µM); the effects are reversed and are much more pronounced, with extended lag-times and a marked reduction in fibre load (Fig. 4f–h). In the case of Aβ(1–40) incubated with 5 µM Cu^2+^ and 300 µM H_2_O_2_, there is no detectable ThT signal even after 250 hours incubation, Fig. 4h. These data indicate conditions which induce dityrosine crosslinking strongly inhibit fibre formation, as detected by ThT fluorescence.

In previous studies we have carefully characterised the impact of Cu^2+^ or H_2_O_2_ alone on Aβ fibre assembly^34,74,75^. In particular, Cu^2+^ ions and H_2_O_2_ do not directly affect ThT as a detector of fibres. It is notable that Cu^2+^ alone accelerates Aβ(1–40) fibre formation with little impact to ThT maximum signal Supplemental Fig S3a^74,75^. While H_2_O_2_ on its own accelerates fibre formation but reduces ThT maximum intensity as previously reported, Supplemental Fig S3b^34^. We were concerned that the presence of Cu^2+^ and H_2_O_2_ might react with the fluorescence dye, ThT, and disrupt the detection of Aβ fibres. However, there is not a gradual loss of signal due to chemical modification of ThT, as the signal is maintained over hundreds of hours, as shown in Fig. 4b,c. Although, the H_2_O_2_ + Cu^2+^ addition to samples in which fibres are pre-formed caused some reduction in ThT intensity over the first few hours, the ThT fluorescence signal is maintained for over 150 hours after this initial reduction (data shown later). We conclude, Cu^2+^ + H_2_O_2_ does not substantially interfere with the ThT dye as fluorescence is observed when fibres are present.

We were also interested in the effect of Cu^2+^ with H_2_O_2_ on the assembly of Aβ(1–42). Similar effects to Aβ(1–40) were observed when using ThT to detect fibre inhibition. However, it is notable that unlike Aβ(1–40), Cu^2+^ ions even in the absence of H_2_O_2_ will cause Aβ(1–42) to be trapped in an oligomer state^75^. So, in the case of Aβ(1–42), it is difficult to differentiate the effects of Cu^2+^ binding alone from dityrosine formation.

### The effect of di-tyrosine formation on fibre morphology

**Figure 5:**
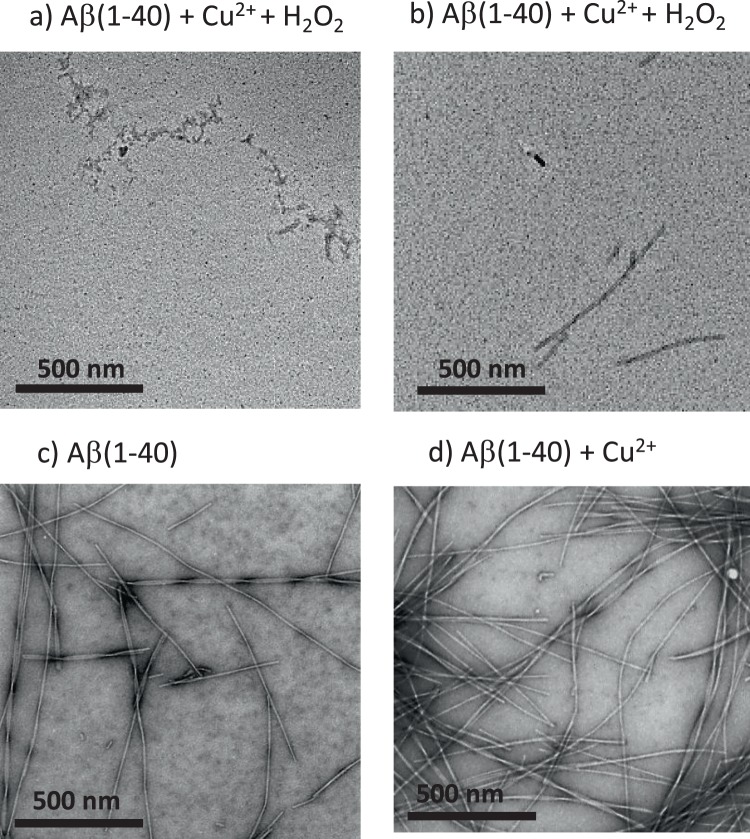
TEM images of Aβ(1–40) assemblies formed with and without H_2_O_2_ + Cu^2+^. Redox cycling of Cu^2+/+^ oxidizes Aβ(1–40) and cause a reduction in both the number, and the maximum length of fibres, while promoting short curvy-linear protofibrils. 10 µM Aβ(1–40) was incubated with (**a**) 5 µM Cu^2+^  + 300 µM H_2_O_2_ for 300 hours, (**b**) 5 µM Cu^2+^  + 300 µM H_2_O_2_ for more than 2 weeks, and also (**c**) Aβ(1–40) in the absence of Cu^2+^ and H_2_O_2_. (**d**) Aβ(1–40) with 5 µM Cu^2+^. All samples contain 100 mM HEPES at pH 7.4 and 160 mM NaCl at 30 °C with intermittent agitation.

To gain further insight into the morphological effects to Aβ(1–40) fibre formation in the presence of the Cu^2+^ and H_2_O_2_ redox system, Aβ assemblies were examined using TEM, Fig. 5. Upon incubation of monomeric Aβ(1–40) with Cu^2+^ + H_2_O_2_ for 300 hours, TEM images taken over numerous grids consistently indicate there to be very few detectable Aβ assemblies. These assemblies do not have a fibrous appearance; they are short “curvy linear” structures typically only 50 nm long and 10 nm thick, Fig. 5a. After further incubation for 2 weeks, these structures were superseded by a limited number of amyloid fibres typically 10 nm thick. The lengths of these structures do not exceed 500 nm, Fig. 5b. These fibres are much less abundant and are shorter in length than Aβ(1–40) fibre generated in the absence of oxidizing Cu + H_2_O_2_, Fig. 5c.

**Figure 6:**
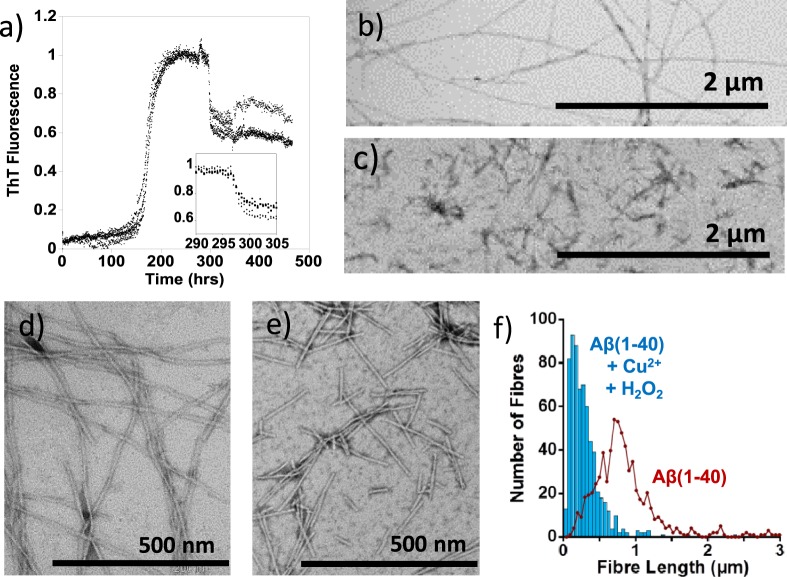
Impact of Cu^2+^ with H_2_O_2_ on preformed Aβ(1–40) fibres. Cu^2+/+^ redox cycling cause a reduction in total fibre mass and causes fibres to fragment into short (<600 nm) Aβ fibres. (**a**) ThT fluorescence monitored over time (normalized to maximum), with 5 µM Cu^2+^ and 300 µM H_2_O_2_ added after 290 hours, causing a 40% reduction in fibre mass within 5 hours (shown in zoomed insert). TEM images show the morphology of Aβ assembly state both before (**b**,**d**), and following (**c**,**e**), an addition of Cu^2+^ and H_2_O_2_ to preformed fibres and further incubation. (**f**) Quantification of fibre length before and after the addition of Cu^2+^  + H_2_O_2_, with more than 600 fibres measured for each condition. Aβ(1–40) (10 µM) was incubated with 100 mM HEPES buffer at pH 7.4, 20 µM ThT and 160 mM NaCl, at 30 °C with intermittent agitation. Scale bar, 1 µm and 500 nm.

*In vivo* Aβ fibres could potentially form first and subsequently be oxidized by redox active Cu^2+^. We therefore studied the effect of the oxidising system on pre-formed amyloid fibres, Fig. 6. Cu^2+^ with H_2_O_2_ was added to pre-formed fibres, causing the ThT signal to drop by 35% over a few hours. This indicates some loss in the total fibre mass, Fig. 6a. The addition of H_2_O_2_ only to preformed fibres has no effect on the ThT signal, it is only when H_2_O_2_ is added to Cu^2+^ bound Aβ(1–40), that we observed a reduction in ThT signal and fragmentation of fibres, as shown in Supplemental Fig S4. This observation is supported by the TEM images, Fig. 6, which shows widespread fragmentation of the preformed fibres. We have quantified the length of Aβ(1–40) fibres before and after the addition of Cu^2+^ + H_2_O_2_. Figure 6f show data for more than 600 fibres for each condition. The Aβ(1–40) fibres imaged exhibit a range of fibre lengths typically more than 800 nm and sometimes many microns in length. In marked contrast, after incubation with Cu^2+^ + H_2_O_2_ the fibres become highly fragmented and are typically 150 nm in length and tend not to exceed 600 nm, Fig. 6f. A combination of the TEM images and the observed reduction of ThT florescence suggest that dityrosine cross-links within pre-formed fibres will cause widespread fragmentation of Aβ fibres. While dityrosine generated from Aβ monomers markedly inhibits fibre formation, and the sparse number of amyloid fibres that do form are also fragmented.

### The impact of a Cu^2+^ with Ascorbate Redox System on Aβ fibrillisation

**Figure 7:**
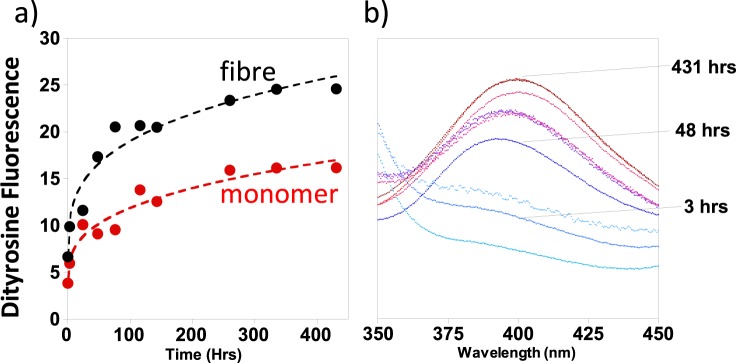
Dityrosine fluorescence of Aβ(1–40) generated with Cu^2+^  + ascorbate. Both Aβ monomer and fibre can form dityrosine. Using the Cu + Ascorbate redox system, Aβ(1–40) fibres form dityrosine more readily. (**a**) Dityrosine fluorescence at 410 nm plotted against time, on incubation of 10 µM Aβ monomer (red), or fibre (black), with 5 µm Cu^2+^ and 50 µM ascorbate. (**b**) Dityrosine fluorescence spectra. Samples contained HEPES buffer (100 mM) at pH 7.4 and NaCl (160 mM).

In order to further understand dityrosine formation within Aβ, we investigated the oxidizing effects of the Cu^2+^  + ascorbate redox system. Formation of dityrosine has been monitored by the appearance of a fluorescence emission at 410 nm. The influence of Cu^2+^ + ascorbate on dityrosine formation appears broadly similar to Cu^2+^ + H_2_O_2_, although the fluorescence signal is shifted by 10 nm to shorter wavelengths. As with the Cu^2+^ + H_2_O_2_ system, we have compared the extent by which Aβ monomers or fibres can be oxidized. The Cu^2+^ + ascorbate system results in dityrosine formation to a greater extent for pre-formed fibres, compared to monomeric Aβ(1–40), Fig. 7. We were surprised by this observation because for the Cu^2+^ + H_2_O_2_ redox system, the opposite behaviour was observed, as previously shown in Fig. 3.

**Figure 8:**
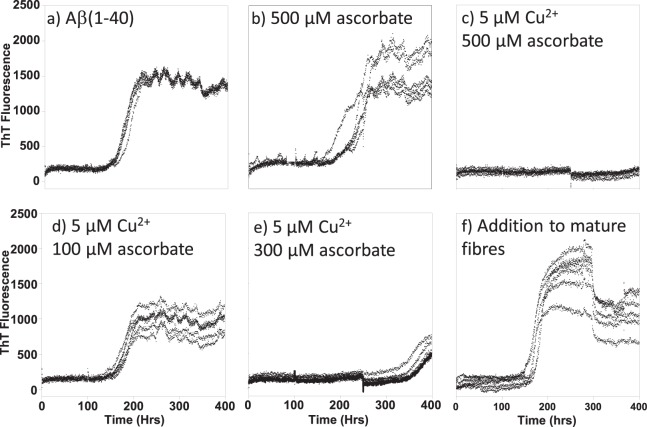
Cu^2+^ with Ascorbate inhibits Aβ(1–40) Fibre Formation. Redox cycling of Cu^2+/+^ oxidizes Aβ and inhibits its formation of fibres, causing extended lag-times and reduced fibre mass. ThT (20 µM) was used to monitor Aβ(1–40) 10 µM fibre formation in the presence of different amounts of Cu^2+^ with ascorbate: (**a**) Ab(1–40) alone; (**b**) 500 µM ascorbate; (**c**) 5 µM Cu^2+^/500 µM ascorbate; (**d**) 5 µM Cu^2+^  + 100 µM ascorbate; (**e**) 5 µM Cu^2+^  + 300 µM ascorbate. (**f**) 5 µM Cu^2+^  + 500 µM ascorbate were added to preformed fibres after 300 hours and a reduction in fibre load occurs within 5 hours. Samples contained HEPES buffer (100 mM) at pH 7.4 and NaCl (160 mM) at 30 °C, under intermittent agitation.

Next we determined how oxidizing Aβ(1–40) using Cu^2+^ with ascorbate affects the ability for Aβ(1–40) monomer to form fibres, as detected by ThT fluorescence dye, Fig. 8. We note that 500 µM of ascorbate, in the absence of Cu^2+^, has little effect on fibre formation, Fig. 8b, while the additional presence of 5 µM Cu^2+^ with 500 µM ascorbate totally inhibits fibrillisation over 400 hours of incubation (Fig. 8c). Incubation of Aβ(1–40) with 5 µM Cu^2+^, but less ascorbate only partially inhibits fibre formation. In particular, Cu^2+^ plus 300 µM ascorbate does not completely inhibit fibre formation but extends the lag-phase to 350 hours, while Cu^2+^ plus 100 µM ascorbate only reduces the ThT maximum signal a little, Fig. 8d,e. Furthermore, the addition of 5 µM Cu^2+^ and 500 µM ascorbate to pre-formed fibres decreases the ThT signal by ~50% within a few hours, Fig. 8f.

**Figure 9:**
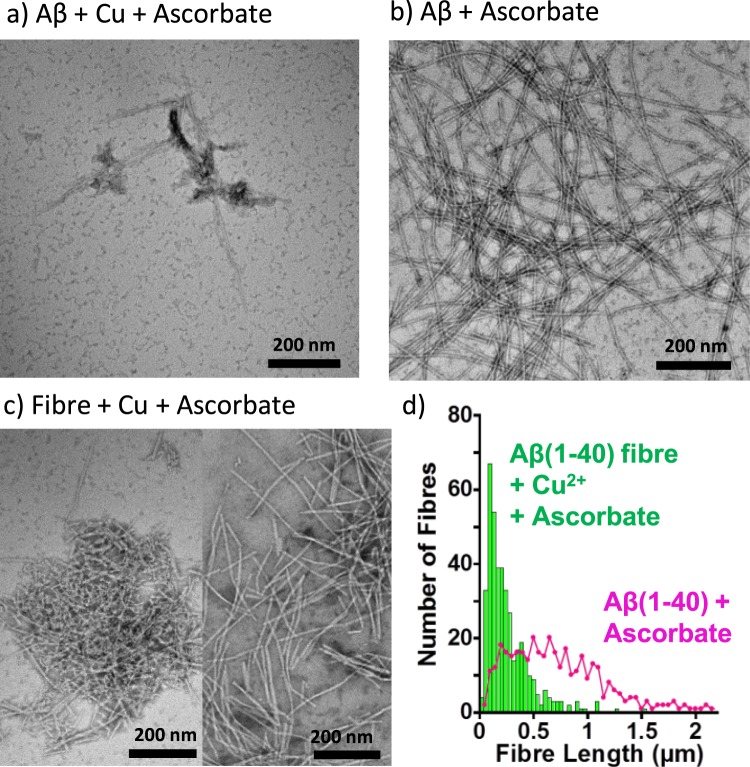
TEM of Aβ(1–40) incubated with Cu^2+^ and ascorbate. Redox cycling of Cu^2+/+^ by ascorbate oxidizes Aβ(1–40) and inhibits fibre formation while promoting short curvy-linear protofibrils. Preformed Aβ(1–40) fibres are fragmented into curvy-linear protofibrils by Cu^2+/+^ with ascorbate. (**a**) Aβ (10 µM) with Cu^2+^ (5 µM) and ascorbate (500 µM). (**b**) 10 µM Ab(1–40) fibres formed with 500 µM ascorbate, but no Cu^2+^ (**c**) Preformed Aβ(1–40) fibres subsequently incubated with 5 µM Cu^2+^ and 500 µM ascorbate. (**d**) Quantification of fibre length before and after the addition of Cu^2+^  + Ascorbate. Data is comprised of more than 300 fibres for each condition.

TEM shows morphological changes similar to those observed with Aβ(1–40) oxidized with Cu^2+^ + H_2_O_2_, Fig. 9. Oxidized Aβ(1–40) monomers formed a very limited number of short fibres with a fragmented appearance, Fig. 9b. Addition of Cu^2+^ with ascorbate to pre-formed fibres causes changes in the morphology of the fibres, which have a more fragmented appearance, shown in Fig. 9c. The distribution of fibre lengths have been quantified for Aβ(1–40) grown in the presence of ascorbate and then upon the addition of Cu(II), Fig. 9d. Median fibre lengths reduce from 700 nm to 150 nm upon oxidation and formation of dityrosine.

## Discussion

A great deal of evidence suggests Cu^2+/+^ redox cycling to be instrumental in oxidative stress in AD^11–13^. A single Cu^2+/+^ ion can bind to one Aβ molecule via its histidine residues to form an Aβ-Cu complex for both Aβ monomers and fibres^25,46,52–55^. The Cu-Aβ complex is redox active and readily produces reactive oxygen species (ROS)^30,56–60^. We chose to oxidize Aβ using Cu^2+^ under reducing/oxidising aerobic conditions, because it is thought to be an important mechanism by which Aβ is oxidized *in vivo*^11–13^. Ascorbate is a ubiquitous molecule^39^, while elevated levels of Cu^2+^ are found at the synapse during depolarization (15–250 µM)^76,77^ and also amyloid plaques^15,23,24^. Cu^2+^ redox cycling causes the generation of hydroxyl radicals which are highly reactive. Cu^2+^ and Cu^+^ form multiple interchangeable tetragonal and linear complexes respectively. These involve at least two of the three His residues within Aβ. Coordination of Cu^2+^ and Cu^+^ to Aβ at residues His6, His13 and His14 places Tyr10 in close proximity to the redox active copper ion^25,46,52–55^. Dityrosine is readily formed as a consequence of nearby ROS generation but other oxidative modifications of Aβ can also occur, including methionine oxidation^15,30,33^ whose impact on fibre formation has recently been characterized^34^. In addition, a small amount of histidine oxidization generates 2-oxo-Histidine^30,31,78^, and some main-chain cleavage can occur^58^. We have shown here that dityrosine crosslinking can occur both with Aβ fibres and Aβ monomer, when in the presence of either the Cu^2+^  + H_2_O_2_, or the Cu^2+^  + ascorbate aerobic redox systems.

We were interested in how the formation of the cross-β-sheet fibre structures might impact the formation of dityrosine cross-links within Aβ fibers. There are numerous studies that reveal atomic level details of the structure of amyloid fibres for both Aβ(1–40) and Aβ(1–42)^79–83^. Deuterium exchange and solid state NMR experiments suggest the in-register β-strands of both Aβ(1–40) and Aβ(1–42) amyloid fibres to occur between residues 13–40, while the N-terminal residues with the Cu^2+/+^ binding site remain unstructured. In particular, the single tyrosine at position 10 has a good deal of flexibility even within amyloid fibres^79–83^. Aβ amyloid fibres exhibit in-register stacking of side-chains from successive Aβ molecules along the length of the fibre. Adjacent Tyr residues are aligned in close proximity to each other, so that the tyrosine residues are sufficiently close to form a covalent link. We hypothesised that this might facilitate tyrosine cross-linking more readily than for the monomer. Alternatively, the position of two adjacent strands might mean cross-linking is less favoured because of the steric consideration of adjacent Tyr residues. Our data of dityrosine formation indicates that with a Cu^2+^ + H_2_O_2_ oxidizing system, Aβ monomer forms dityrosine more readily than for Aβ fibres, although both monomer and fibres will form dityrosine. The opposite behaviour is observed when Aβ is oxidized with the Cu^2+^ + ascorbate system with a slight preference for dityrosine formation within Aβ fibres. It may be that the hydrophobic side-chains are buried in the fibre, allowing ascorbic acid to interact with the Cu-Aβ more readily. Importantly, this data suggests that *in vivo* dityrosine can continue to be generated even once Aβ plaques are formed. This is of particular relevance *in vivo*, as redox active Cu^2+^ is concentrated in plaques bound to Aβ^15,23–25,46,49^. We considered the possibility that the dityrosine crosslinking might occur between different fibres, forming a covalently linked mesh of many fibres, and certainly the data is consistent with this. However, it is important to note that the intensity of the dityrosine fluorescence signal indicates that the majority (>50%) of the Tyr residues have formed covalent dimers, which suggests that most of these occur in adjacent β-strands within a single amyloid fibre. We note that dityrosine formation can only result in covalent dimers of Aβ, because Aβ contains only a single Tyr. The large oligomers formed^17,29^ must be stabilised by non-covalent interactions, in addition to covalent dimer formation.

We also aimed to determine if dityrosine formation might accelerate or inhibit the rate of fibre formation. The process by which Aβ monomer assembles into amyloid fibres includes a kinetically slow step which involves the formation of a small nucleating “seed”. Once a templating oligomer is formed, Aβ monomers are rapidly recruited to extend the fibre length. *In vitro* this process is reported as a sigmodal fibre growth curve; this includes a lag-phase, and a rapid elongation phase followed by a plateau once equilibrium is reached. The fibre-nucleating form of Aβ could be a misfolded monomer of Aβ, or a small oligomer. It is also suggested the nucleating form of Aβ could be a simple dimer. A dityrosine dimer might result in rapid acceleration of fibre production. Indeed, this seems to be the case for dityrosine formation within Parkinson’s disease linked α-synuclein, which causes acceleration of α-synuclein fibre formation^69,70^. However, we show here that this is not the case for Aβ; indeed dityrosine formation strongly inhibits Aβ fibre formation. Others have generated Aβ covalent dimers of dityrosine Aβ using solid-phase synthesis^63^, or the use of horse radish peroxidase with H_2_O_2_^68^, or Cu^2+^  + H_2_O_2_ to oxidize the tyrosine^66^. These methods of dityrosine formation also cause inhibition of fibre formation^63,66,68^. We can infer from our *in vitro* studies that dityrosine formation is unlikely to be a trigger for rapid fibre formation *in vivo*.

Our studies also indicate that even after fibres are formed, Cu^2+^ in the presence of a physiological reductant such as ascorbate, can form dityrosine and will also cause widespread fragmentation of preformed fibres. This is important, as Cu^2+^ is directly bound to Aβ within AD plaques and will be redox active. Dityrosine cross-linking can increase the non-degradability of mature fibres once formed, perhaps forming a network of cross-linked fragmented fibres.

More generally, there is much interest in how Aβ dimer formation impacts the assembly of Aβ into larger oligomers and fibres. Fibre formation kinetics have been studied for a range of other covalent Aβ dimers summarized in Table 1^63–65,68,84,85^. In one study, the alanine at position 2 was replaced by cysteine, and a covalent disulphide bond was introduced between two Aβ molecules at position 2^84^. Interestingly, the assemblies that were formed with Aβ(Ala2Cys)-dimer have a strikingly similar appearance to the assemblies formed for the dityrosine Aβ-dimer reported here, producing short fragmented fibre assemblies^84^. In contrast a Cys dimer for a Aβ(Ser26Cys) produced very different assemblies, reducing the lag-time to nucleate fibre formation^64,68^. Furthermore, Aβ dimers that are also linked at position 10 with a shorter 3-carbon alkane chain, behave differently from dityrosine and exhibit accelerated fibre formation^63,65^. Conversely, dimers formed at Lys16 also inhibit fibre formation^85^. The ability to accelerate or inhibit fibre formation is sensitive not only to the position of the dimerization in the sequence but also to the geometry of the linker used to dimerise Aβ, Table 1.

**Table 1:** Aβ covalent dimers and their fibre formation kinetics.

Aβ dimers	Fibre formation	Reference
Aβ S26C	Accelerated nucleation	^64, 68^
Aβ K16QK	Extended lag-time	^85^
Aβ A2C	Extended lag-time	^84^
Aβ Y10A-linker	Accelerated nucleation	^63, 65^
Aβ diY10-(synthetic)	Extended lag-time	^63^
Aβ diY10-(Cu^2+^ + H_2_O_2_)	Extended lag-time	^66^, (this study)
Aβ diY10-(Cu^2+^ + Ascorbate)	Extended lag-time	(this study)
Aβ diY10-(peroxidase)	Extended lag-time	^68^

The relationship between dityrosine formation, Aβ synaptotoxicity, and AD pathology is yet to be resolved. The soluble Aβ oligomers extracted from AD patients’ brains suggest that Aβ dimers are the smallest cytotoxic species^4,10^. Indeed, it is suggested that dityrosine formation is key to cytotoxicity^17,29,40,63,68^, supported by an observed ability for dityrosine dimers to cause disruption to biomimetic lipid vesicles^66^. This study suggests that Cu^2+^ redox cycling does not promote amyloid fibres, but rather it traps Aβ in a potentially more cytotoxic oligomeric and fragmented assembly state.

To conclude, covalent dimers of Aβ formed by Tyr10 cross-linking have a profound impact on the kinetics of fibre formation, as well as the morphology of the assemblies. It is also apparent that the dityrosine observed *ex vivo* in plaques^17,32^, can be generated after amyloid fibres are formed. Rather than dityrosine formation accelerating fibre formation, this oxidation process is likely to cause cross-linking and fragmentation of pre-formed fibres within plaques. This will have a significant effect on Aβ cytotoxicity, as well as the morphology and clearance of plaques.

## Materials and Methods

### Solubilisation of Amyloid-β

Aβ(1–40) and Aβ(1–42) (from Cambridge Research Biochemicals) was dissolved at 0.7 mg/mL pH 10 and kept at 4 °C with gentle rocking for 8 hours, this has been found to be an effective solubilization protocol^86,87^. Aβ(1–42) in particular required purification via SEC to obtain a single elution peak. This generated predominantly seed-free Aβ stock, based on a clear consistent lag-phase observed via ThT fluorescence, with a lack of detectable assembles in TEM images and a single elution peak in size exclusion chromatography. A single batch of Aβ was used for any set of experiments, so that controls were directly comparable. All other chemicals were purchased from Sigma.

### Dityrosine Formation

Typically dityrosine was produced under aerobic conditions using Aβ (10 µM) and substochiometric Cu^2+^ (5 µM CuCl_2_). Fenton reactions require the presence of a reductant, here we used between 50 µM −1.6 mM H_2_O_2_ or between 50–500 µM ascorbate. Solutions contained HEPES buffer (100 mM) at pH 7.4 and NaCl (160 mM). The cycling of copper between its two oxidation states results in the production of hydroxyl radicals which will generate a tyrosine radical, which then go on and form dityrosine with a second tyrosine sidechain, see Fig. 1.

### UV and Fluorescence detection of Dityrosine

Dityrosine has a fluorescence maximum at 410 nm, using an excitation wavelength of 310 nm. Dityrosine emission was monitored between 300 and 500 nm using a 1 cm quartz cuvette (Hellma) and a Hitachi F-2500 fluorescence spectrophotometer.

UV absorbance was also used to monitor dityrosine production of the covalent dimer by monitoring the absorbance spectrum at 315 nm with an extinction coefficient of 5,000 M^−1^ cm^−1^ ^71,72^, using a 1 cm quartz cuvette (Hellma). Difference spectra, with non-oxidized Aβ subtracted from each spectrum are presented.

### Fibre Growth Assay

Fibres were generated by incubation of Aβ(1–40) or Aβ(1–42) at 10 µM, HEPES buffer (100 mM) at pH 7.4 with NaCl (160 mM) and Thioflavin (ThT) (20 µM) at 30 °C. BMG-Galaxy and Omega fluoro-star fluorescence 96-well plate readers were used to monitor fibre formation with mild agitation (60 seconds every 30 minutes). The binding of ThT to amyloid fibres was used to monitor the kinetics of Aβ fibre growth. When bound to amyloid fibres, ThT fluoresces at a maximum of 489 nm, the intensity of which is directly related to the concentration of Aβ fibres present^88^. By exciting at 440 nm and measuring the fluorescence at 490 nm, fibre formation can be followed over time^89,90^. Cautious interpretation of ThT fluorescence as a quantitative measure of fibre mass is needed^89^, as not all Aβ assemblies fluoresce with ThT. In particular, the ThT fluorescence with prefibrillar oligomers and protofibrils of Aβ are typically very weak^88^. Furthermore it may be possible that the intensity of fluorescence may vary slightly for different fibre morphologies.

Conversion of Aβ monomer to fibre follows a characteristic sigmoidal growth curve, which has a lag-phase (nucleation) and a growth-phase (elongation). The lag-phase involves the formation of an increasing number of small nucleating assemblies. The number of individual assemblies (rather than assembly mass) can increase by both primary and secondary nucleation and also fragmentation. The growth-phase (elongation) is dominated by the addition of Aβ monomers on to the ends of growing fibres which leads to rapid increases in fibre mass (and ThT fluorescence)^89^. Some important empirical parameters were obtained from the fibre growth curves, including the time needed to reach half-maximal ThT intensity (t_50_), the apparent fibre elongation rate (k_app_) and the lag-time to nucleate fibres (t_lag_)^91^.

### Transmission Electron Microscopy (TEM)

Glow-discharged carbon-coated 300-mesh copper grids, purchased from SPI, were prepared using the droplet method, where 10 µl aliquots of samples from the fibre growth assay were adsorbed for 1 min and blotted with filter paper. After rinsing with deionized water (10 µl for 1 minute) and blotting, samples were placed onto a drop of 2.0% phosphotungstic acid (PTA) (purchased from Sigma), (10 µl for 1 minute), blotted, rinsed and air-dried. Images were recorded on a JEOL JEM-1230 electron microscope operated at 80 keV. Fibre lengths were measured manually for each preparation using image-J software.

### SDS-PAGE

Aβ samples (100 µM) were diluted 1:1 into Biorad SDS Laemmli loading buffer and boiled for 10 minutes before running at 200 V for 1 hour on a 15% SDS polyacrylamide gel (10 µl per well). Gels were then stained using Coomassie Brilliant Blue R-250 (Sigma-Aldrich Company Ltd., UK). SDS-PAGE was performed on BioRad Mini-Protean electrophoresis cells. Gel-band lane profiles were generated using image-J and band intensities were quantified using Biorad Image Lab 6.0.1 gel analysis software.

### Size Exclusion Chromatography (SEC)

Seed-free Aβ stock was generated using SEC. The samples were loaded on to a Superdex prep grade S75/200 HR 10/30 column (GE Healthcare) using ÄKTA system (GE Healthcare). The column was pre-equilibrated with buffer and run at 0.5 ml/min. Then Aβ samples (20 µM) of 200 µl were loaded on to the column with a flow rate of 0.5 ml/min.

## Electronic supplementary material

Supplementary Information
