# Supplementary material for: Copper Redox Cycling Inhibits Aβ Fibre Formation and Promotes Fibre Fragmentation, while Generating a Dityrosine Aβ Dimer
Source: Sci Rep. 2018 Nov 1;8:16190. doi: 10.1038/s41598-018-33935-5 (PMC6212427; doi:10.1038/s41598-018-33935-5)
Supplement: Supplementary file 1 — Supplementary Information [file 41598_2018_33935_MOESM1_ESM.pdf]

Copper Redox Cycling Inhibits Aβ Fibre Formation and Promotes Fibre Fragmentation, while Generating a Dityrosine Aβ Dimer

Miao Gu, David C. Bode and John H. Viles

Supplemental Information

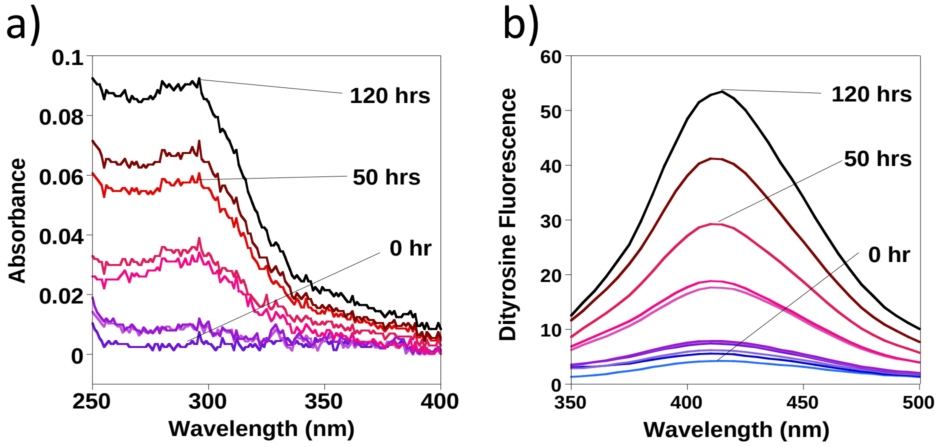

Supplemental Figure S1: Monitoring Dityrosine formation. Comparison of UV absorption and fluorescence spectra of Aβ(1-16) in a Cu+H_2O_2 redox system. Difference UV absorption spectra (a), and fluorescence spectrum excitation at 310 nm (b). 50 μM Aβ(1-16) was incubated with 10 μM Cu^{2+} and 1000 μM H_2O_2, with 100 mM HEPES buffer pH 7.4 over 120 hours.

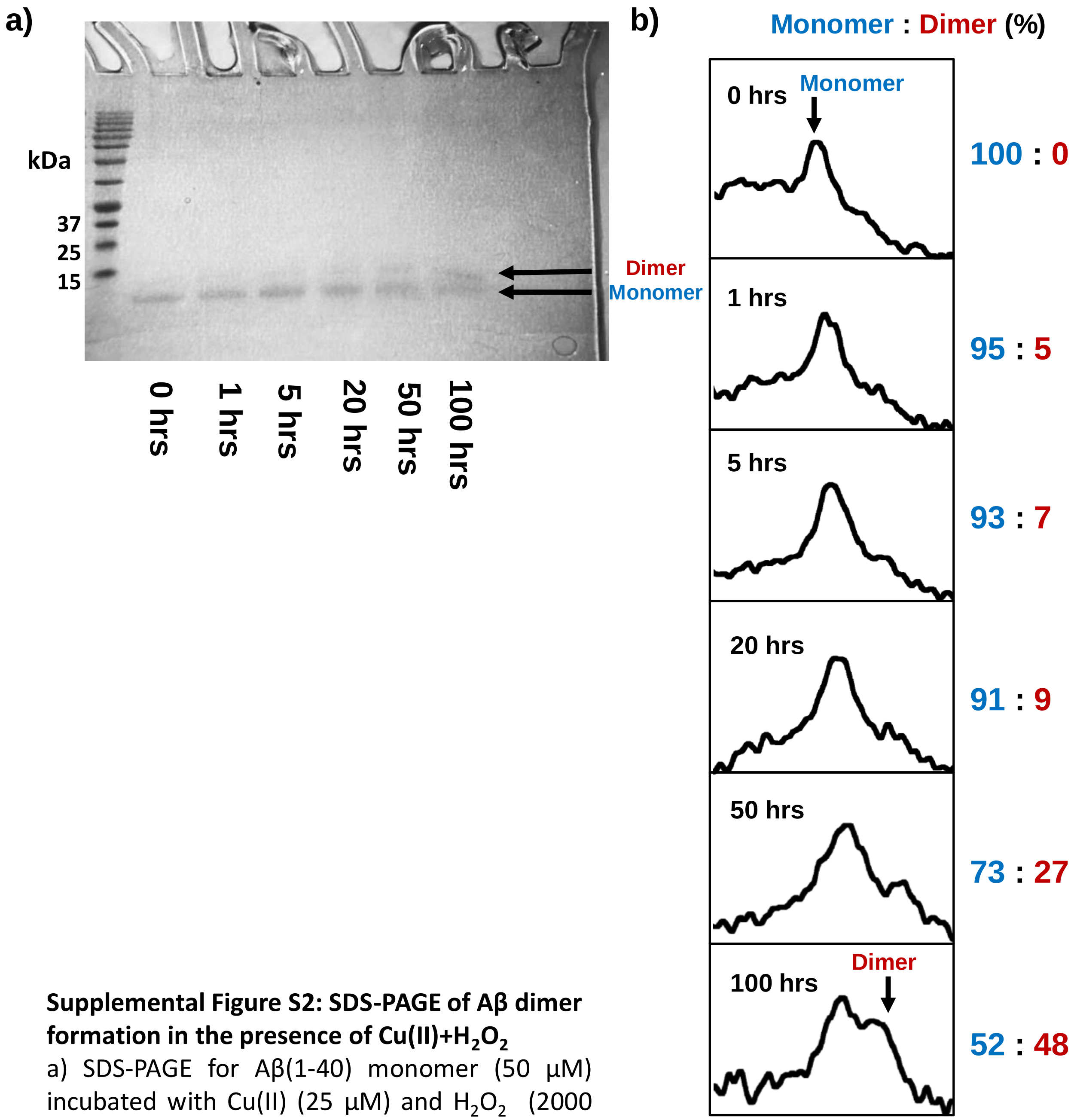

Supplemental Figure S2: SDS-PAGE of Aβ dimer formation in the presence of Cu(II)+H_2O_2

a) SDS-PAGE for Aβ(1-40) monomer (50 μM) incubated with Cu(II) (25 μM) and H_2O_2 (2000 μM) over 100 hours.

b) Gel-band intensities measured at each time point reveals that half of the Aβ monomer is converted to a dimer within 100 hours incubation.

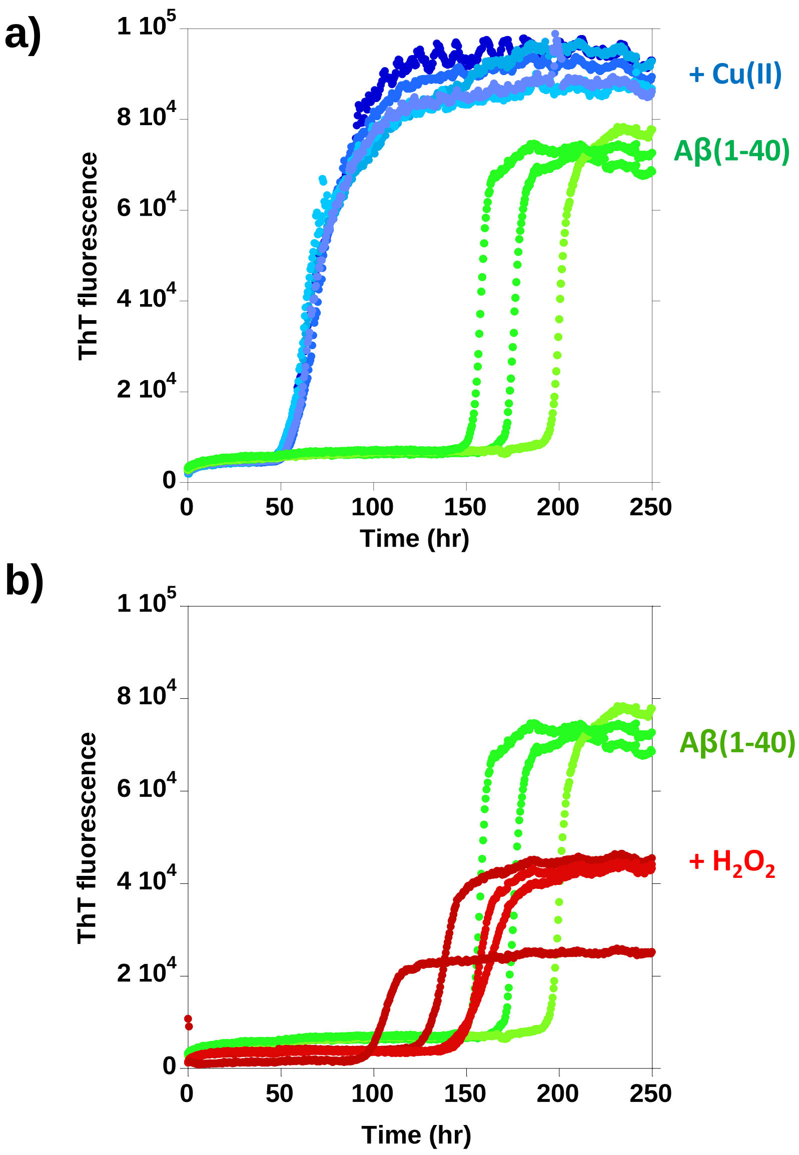

Supplemental Figure S3. The effect of Cu(II) and H_2O_2 on Aβ(1-40) in the absence of radical production and dityrosine formation.

ThT fluorescence was used to monitor Aβ fibre formation. **a)** 10 µM Aβ(1-40) (green) + 5 µM Cu^{2+} (blue). The presence of sub-stoichiometric Cu(II) alone accelerates Aβ(1-40) fibre formation, typically halving the lag-times.

b) H_2O_2 also accelerates fibre formation a little, and reduces the total amount of fibre formed. 10 µM Aβ(1-40) (green) + 300 µM H_2O_2 (red).

Aβ(1-40), 10 µM, was incubated with 100 mM HEPES pH 7.4, 20 µM ThT and 160 mM NaCl at 30°C with intermittent agitation.

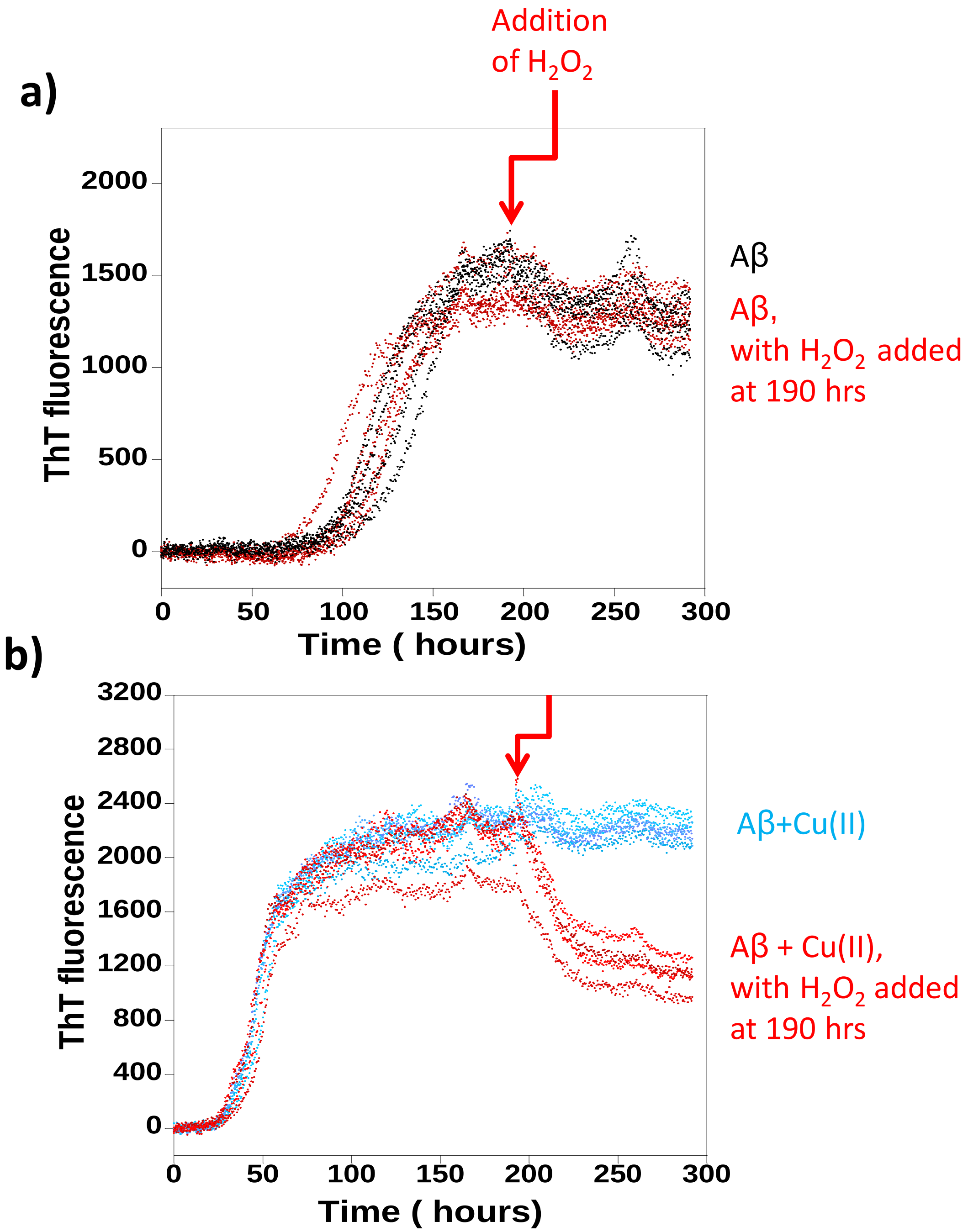

Supplemental Figure S4: Addition of H_2O_2 to preformed Aβ fibres or Aβ+Cu fibres
a) Addition of H_2O_2 to Aβ after 190 hrs has no impact on ThT fluorescence intensity.
b) Addition of H_2O_2 to Aβ with Cu(II) after 190 hrs halves the ThT fluorescence intensity within 20 hours. Aβ(1-40), 10 µM, was incubated with 100 mM HEPES pH 7.4, 20 µM ThT and 160 mM NaCl at 30°C with intermittent agitation.
